# Autophagy signaling in hypertrophied muscles of diabetic and control rats

**DOI:** 10.1002/2211-5463.13677

**Published:** 2023-07-26

**Authors:** Maria V. M. Scervino, Marco A. S. Fortes, Kaio F. Vitzel, Diego R. de Souza, Gilson M. Murata, Giovanna O. Santana, Eliane B. da Silva, Adriana C. Levada‐Pires, Wilson M. T. Kuwabara, Tatiana C. A. Loureiro, Rui Curi

**Affiliations:** ^1^ Instituto de Ciências da Atividade Física e Esporte (ICAFE) Universidade Cruzeiro do Sul São Paulo Brazil; ^2^ Departmento de Fisiologia e Biofísica, Instituto de Ciências Biomédicas Universidade de São Paulo Brazil; ^3^ Departmento de Nutrição Centro Universitário Avantis Balneário Camburiú Brazil; ^4^ School of Health Sciences, College of Health Massey University Auckland New Zealand; ^5^ Departamento de Projetos de Pesquisa e Ensino Escola de Educação Física da Polícia Militar do Estado de São Paulo Brazil

**Keywords:** autolysosome, autophagosome, autophagy‐related genes, hyperglycemia, protein degradation

## Abstract

Autophagy plays a vital role in cell homeostasis by eliminating nonfunctional components and promoting cell survival. Here, we examined the levels of autophagy signaling proteins after 7 days of overload hypertrophy in the extensor digitorum longus (EDL) and soleus muscles of control and diabetic rats. We compared control and 3‐day streptozotocin‐induced diabetic rats, an experimental model for type 1 diabetes mellitus (T1DM). EDL muscles showed increased levels of basal autophagy signaling proteins. The diabetic state did not affect the extent of overload‐induced hypertrophy or the levels of autophagy signaling proteins (p‐ULK1, Beclin‐1, Atg5, Atg12‐5, Atg7, Atg3, LC3‐I and II, and p62) in either muscle. The p‐ULK‐1, Beclin‐1, and p62 protein expression levels were higher in the EDL muscle than in the soleus before the hypertrophic stimulus. On the contrary, the soleus muscle exhibited increased autophagic signaling after overload‐induced hypertrophy, with increases in Beclin‐1, Atg5, Atg12‐5, Atg7, Atg3, and LC3‐I expression in the control and diabetic groups, in addition to p‐ULK‐1 in the control groups. After hypertrophy, Beclin‐1 and Atg5 levels increased in the EDL muscle of both groups, while p‐ULK1 and LC3‐I increased in the control group. In conclusion, the baseline EDL muscle exhibited higher autophagy than the soleus muscle. Although TDM1 promotes skeletal muscle mass loss and strength reduction, it did not significantly alter the extent of overload‐induced hypertrophy and autophagy signaling proteins in EDL and soleus muscles, with the two groups exhibiting different patterns of autophagy activation.

## Graphical abstract

We analyzed autophagy proteins in extensor digitorum longus (EDL) and soleus muscles of control and diabetic rats after 7 days of hypertrophy. Basal autophagy proteins were higher in EDL. Diabetes did not affect the hypertrophy or autophagy in either muscle. Soleus showed increased autophagy signaling after hypertrophy. Overall, diabetes had no significant effect on hypertrophy or autophagy in both muscles.

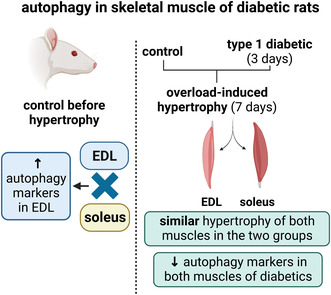

**Abbreviations**
AMPK: protein kinase activated by AMP
Atg: autophagy‐related protein
Bcl‐2: B‐cell lymphoma 2
CL: contralateral
CTRL: control
DM: diabetic
EDL: extensor digitorum longus
H: hypertrophied
LC3: light chain 3
SD: standard deviation
SQSTM1: sequestosome 1
T1DM: type 1 diabetes
ULK‐1: Unc‐51 like autophagy activating kinase
UPS: ubiquitin‐proteasome system

Skeletal muscle accounts for around 40% of body weight in adults and is critical for maintaining blood glucose levels [1]. Type 1 diabetes (T1DM) causes muscle mass loss and weakness due to high proteolysis [2, 3]. Although proteolytic activity threatens long‐term muscle health, proteostasis is crucial in maintaining skeletal muscle functioning [4, 5].

The two best‐known proteolytic systems in skeletal muscle are the ubiquitin‐proteasome (UPS) and autophagy [6, 7]. During autophagy, misfolded/aggregated proteins or damaged organelles are engulfed and degraded in a double membrane that becomes an autophagosome that fuses with lysosomes forming the autolysosome, which exports amino acids and other byproducts to the cytoplasm [8, 9]. Compared with other tissues, skeletal muscle is susceptible to defective autophagy [10, 11]. Upregulation or downregulation of autophagy leads to muscle wasting and weakness [12, 13, 14, 15] and may play a critical role in skeletal muscle mass loss and gain in people with diabetes. Interestingly, while proteolysis and muscle wasting are triggered at the onset of diabetes [16], rats in both the early stage of diabetes (3 days) and after chronic diabetes (30 days) exhibit the same relative response to short‐term (7 days) and long‐term (30 days) overload‐induced skeletal muscle hypertrophy as normoglycemic controls [17, 18].

Herein, we compared changes in autophagy signaling protein levels after 7 days of overload‐induced hypertrophy in skeletal muscles with a predominance of glycolytic extensor digitorum longus (EDL) or oxidative (soleus) fibers in control and diabetic (3 days after streptozotocin induction) rats. We chose a 7‐day experimental period because compensatory overload coincides with high activation of the protein synthesis signaling pathway, which returns to baseline levels after 30 days of hypertrophy [17].

The main objectives of the present study were: (1) to investigate whether seven‐day compensatory overload induces hypertrophy of EDL and soleus muscles in control and diabetic rats in the same magnitude and (2) to explore whether there is a difference in autophagic signaling protein levels between EDL and soleus muscles in control and diabetic rats before or after overload‐induced hypertrophy.

## Materials and methods

### Ethics approval

We used experimental procedures approved by the Ethics Committee for Animal Experimentation of the Institute of Biomedical Sciences at the University of São Paulo (ICB‐USP). Experiments were conducted in accordance with the Guide for the Care and Use of Laboratory Animals (Institute of Laboratory Animal Resources, National Academy of Sciences, Washington DC) and the Brazilian College of Animal Experimentation (COBEA). The protocol is registered under No. 23, page 16, in Book 03 of ICB‐USP for the experimental use of animals.

### Animals

**Fig. 1:**
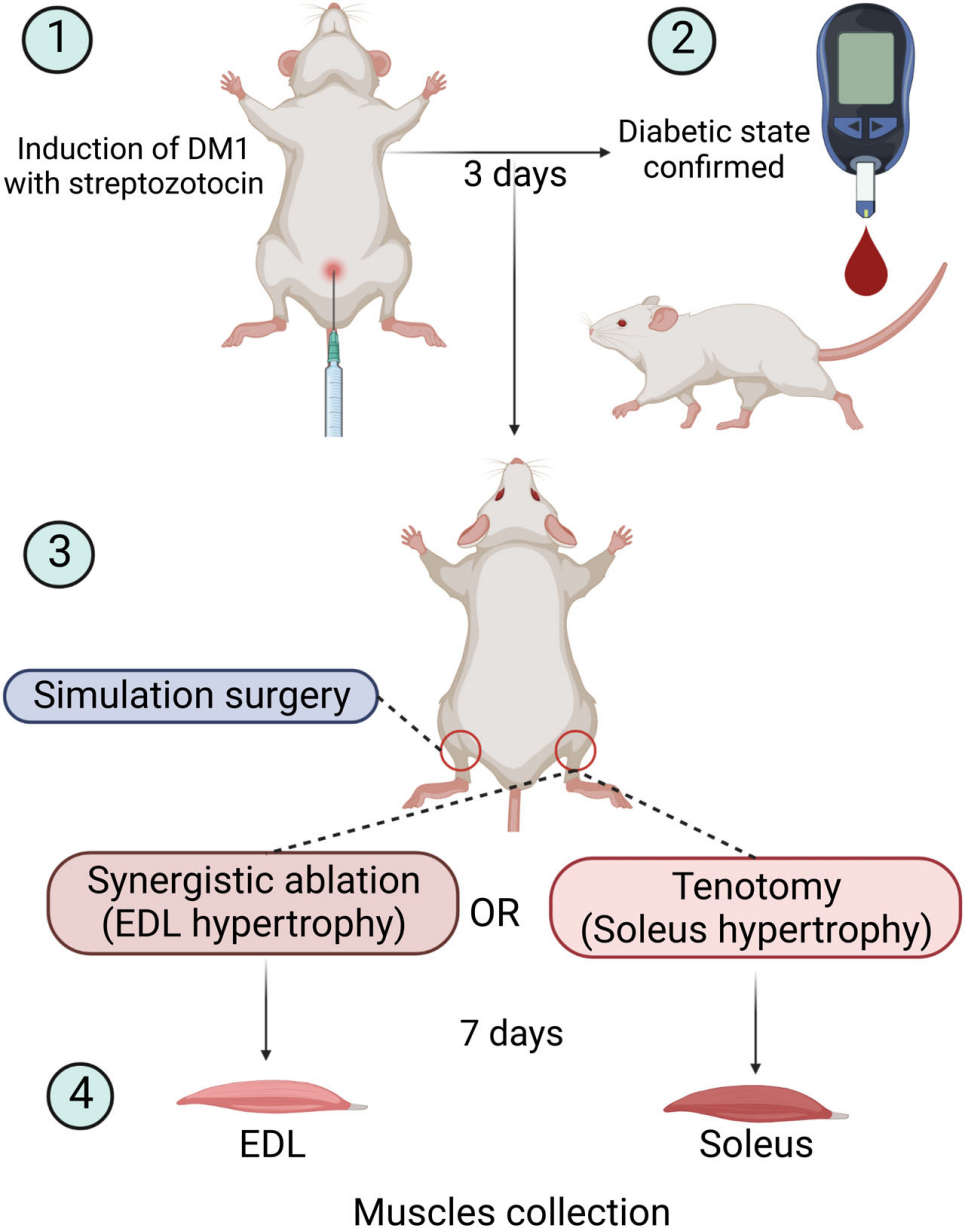
Experimental protocol for induction of diabetic state and overload‐induced hypertrophy of the extensor digitorum longus (EDL) and soleus muscles.

We used 48 eight‐week‐old male Wistar rats (200 ± 50 g) from the ICB‐USP facility. Three rats were housed in each cage and maintained in a room with a 12‐h/12‐h light/dark cycle at 22 °C. Throughout the protocol, the animals had free access to water and standard rodent chow (Nuvilab CR‐1, Quimtia, S/A, Colombo, Brazil) containing 22.5% protein, 55% carbohydrates, and 4.5% fat. We subjected diabetic and control rats to the tibialis anterior muscle ablation for EDL hypertrophy or tenotomy of the gastrocnemius muscle for soleus hypertrophy [17, 18]. After 7 days, the rats were euthanized in a CO_2_‐filled chamber, and EDL and soleus muscles were collected for analysis (Fig. 1).

### Type 1 diabetes mellitus induction

T1DM was induced by a single intravenous injection of streptozotocin (65 mg·kg^−1^ body weight) dissolved in citrate buffer, pH 4.2 [19]. Control animals received an equivalent volume of citrate buffer by the same route [17, 18]. Seventy‐two hours after the streptozotocin injection, blood was drawn from the tail, and glucose concentration was measured with a glucometer (Roche Diagnostics Corporation, Indianapolis, IN, USA). Rats with blood glucose levels above 200 mg·dL^−1^ (11.1 mmol·L^−1^) were considered diabetic [20].

### Synergistic muscle ablation and tenotomy surgeries

Three days after diabetes induction, rats were anesthetized with ketamine and xylazine (i.p. injection of 90 and 10 mg·kg^−1^ body weight, respectively) for surgery. For the ablation of the tibialis anterior muscle, an incision was made in the anterior portion of the animal's left hind paw, exposing the tibialis muscle, which was isolated and removed completely [17, 18, 21, 22].

The gastrocnemius muscle tenotomy was performed by making a longitudinal incision in the posterior portion of the animal's left hind paw, exposing the gastrocnemius and plantar muscles, which had the muscle fascia removed and the distal tendons isolated and sectioned [17, 18, 23, 24].

In both surgeries, a sham operation was performed on the right paw, where the fascia was divulged, but the tendon was not sectioned [25, 26, 27, 28]. The left paws in which the ablation or tenotomy surgeries were performed are called hypertrophied (H), and the right paws in which only the incision and divulsion of the fascia were made are referred to as contralateral (CL). The unilateral operations allow a pairwise comparison between the CL muscles subjected to sham operation and the overloaded muscles. This approach avoids inaccuracies due to using different animals and the systemic modifications this protocol may cause [29, 30].

After 7 days of overload [18, 31], the rats were euthanized by CO_2_ inhalation, and the H and CL EDL or soleus muscles were collected (Fig. 1).

### Western blotting assays of phospho‐ULK‐1, Beclin‐1, Atg5, Atg12‐5, Atg7, Atg3, LC3I/II, and p62/SQSTM1

The phospho‐ULK1 (unc‐51‐like autophagy activating kinase 1), Beclin‐1, Atgs (autophagy‐related proteins) 5, 12–5, 7, and 3, LC3 I and II, and p62/SQSTM1 (sequestosome 1) proteins were chosen for detection because they are proteins present in each step of the autophagy process and act from beginning to end. Radioimmunoprecipitation assay (RIPA) buffer (Thermo Fisher Scientific, Waltham, MA, USA), composed of 50 mm Tris HCl, 150 mm NaCl, 1.0% (v/v) NP‐40, 0.5% (w/v) Sodium Deoxycholate, 1.0 mm EDTA, 0.1% (w/v) SDS, and 0.01% (w/v) sodium azide at pH 7.4, supplemented with phenylmethylsulfonyl fluoride (PMSF; Thermo Fisher Scientific) and SIGMAFAST protease inhibitor (Merk Group, Darmstadt, Germany), was used for protein extraction. The EDL and soleus muscles were homogenized in the protein extraction buffer (500 μL) using a Polytron (Polytron Brinkman, Westbury, NY, USA). After grinding, the samples were placed on ice for 15 min and vortexed in 5‐min intervals. The homogenates were centrifuged at 12 000 ***g*** for 20 min at 4 °C to remove tissue fragments. The supernatants were stored in a −80 °C freezer.

The Pierce BCA Protein Assay Kit (Thermo Fisher Scientific) was used to quantify protein concentrations in supernatants. Samples were mixed with 2× Laemmli Sample Buffer (Bio‐Rad, Hercules, CA, USA) supplemented with 2‐Mercaptoethanol as the reducing agent, added to the buffer before mixing with the samples, and boiled at 95 °C for 5 min. We routinely separated 30 μg of total protein by SDS/PAGE using gels cast from Tris‐Glycine eXtended (TGX) Fast Cast acrylamide solution (Bio‐Rad) and polymerized with 10% ammonium persulfate (APS; Merck) and tetramethylethylenediamine (TEMED; Bio‐Rad) [32]. Proteins were transferred to nitrocellulose membranes using the Trans‐Blot Turbo Transfer System (Bio‐Rad). Primary and secondary antibodies were purchased from Cell Signaling Technology (Danvers, MA, USA) and distributed by Uniscience (São Paulo, Brazil). Membranes were probed with the following primary antibodies: anti‐Phospho‐ULK1 (Ser757, cat. no. 14202), anti‐Beclin‐1 (cat. no. 3738), anti‐Atg5 (cat. no. 12994), anti‐Atg12‐5 (cat. no. 4180), anti‐Atg7 (cat. no. 2631), anti‐Atg3 (cat. no. 3415), anti‐LC3A/B (cat. no. 12741), and anti‐p62/SQSTM1 (cat. no. 5114). All primary antibodies were diluted at 1 : 1000. An anti‐rabbit IgG antibody (cat. no. 7074) diluted at 1 : 5000 was used as the secondary antibody. Membranes were developed using ECL reagent, and images were captured with an Amersham Imager 600UV (Amersham Biosciences, Little Chalfont, UK) after 30–90 s of exposure. The image j v 1.46 software (NIH, Bethesda, MD, USA) was used to quantify the chemiluminescent protein bands.

Commonly used housekeeping protein levels vary in cells and tissues depending on experimental conditions. For example, significant variations in the levels of five housekeeping proteins (GAPDH, β‐actin, α‐tubulin, ɣ‐tubulin, and α‐actinin) were found to be differentially expressed in STZ‐induced diabetes and skeletal muscle hypertrophy models. In these situations, Ponceau S staining is more accurate for quantifying protein loading than the housekeeping proteins tested [33, 34, 35]. Therefore, our findings were normalized to the pool of samples and total protein content as determined by Ponceau S staining [17, 18, 33]. Results are expressed relative to the CL control muscle.

### Statistical analysis

Data are presented as the mean ± standard error of the mean (SEM) and were analyzed first with the Shapiro–Wilk normality test and then with Student's *t*‐test (for comparison between two groups) or two‐way ANOVA (for comparison between three or more groups). The Bonferroni post‐test was used to compare contralateral and hypertrophied muscles of the same group and contralateral and contralateral muscles and hypertrophied and hypertrophied muscles of different groups. As indicated in the text and figure legends, ANOVA was used only for comparing diabetic and control rats, considering both contralateral and hypertrophied muscles and the comparisons between diabetic and control hypertrophied muscles and diabetic and control contralateral muscles. Grubb's test was used to exclude outliers. Differences between results were considered significant for p values < 0.05. All results were analyzed using graphpad prism 5.0 statistical software (GraphPad Software, San Diego, CA, USA).

## Results

**Fig. 2:**
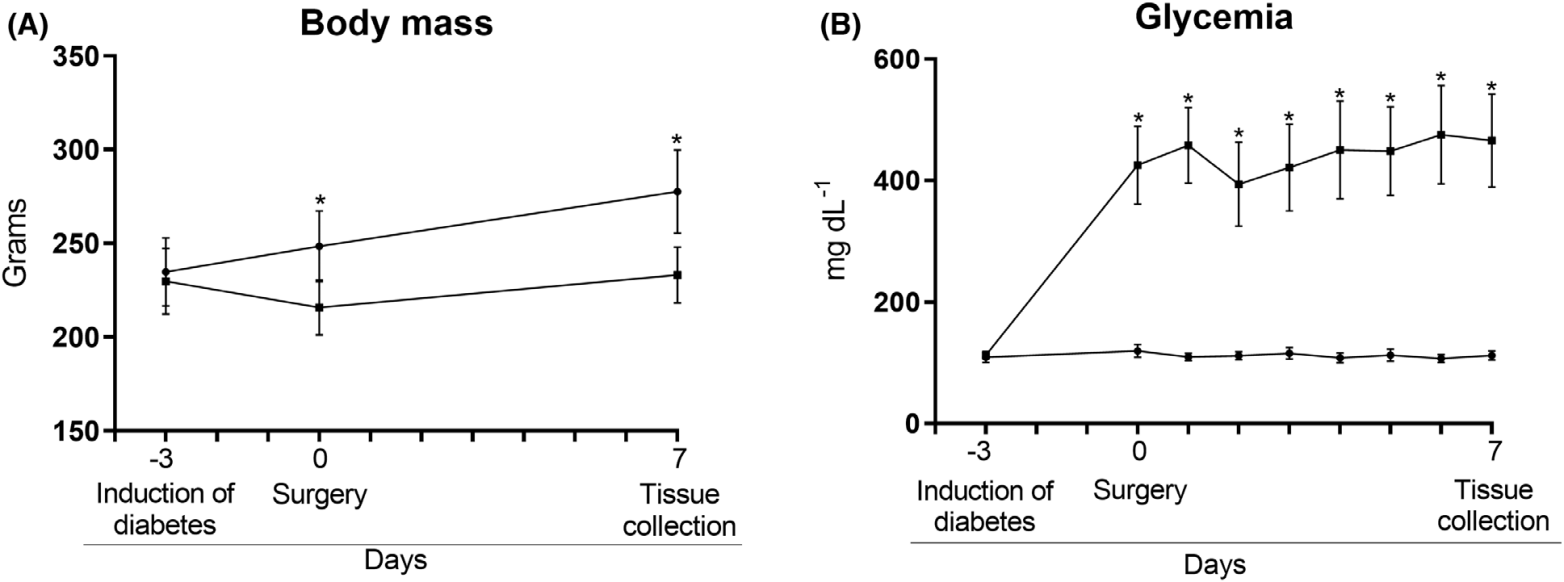
Body mass (A) and glycemia (B) of the control and diabetic groups. −3 = day of diabetes induction via injection of streptozotocin (diabetic group) or injection of citrate buffer (control group); 0 = day of skeletal muscle surgery in both groups; 7 = day of muscle collection for analysis. *P < 0.001 as indicated by two‐way ANOVA and subsequent Bonferroni post‐test. Values are expressed as mean ± SD. Number of animals used in each group: control group (CTRL) = 20; diabetic group (DM) = 16.

During the 7‐day experimental period, the body mass of control animals increased by 42.8 ± 7.1 g but remained unchanged in the diabetic rats (Fig. 2, panel A). The blood glucose levels of diabetic rats increased fourfold (Fig. 2, panel B).

**Fig. 3:**
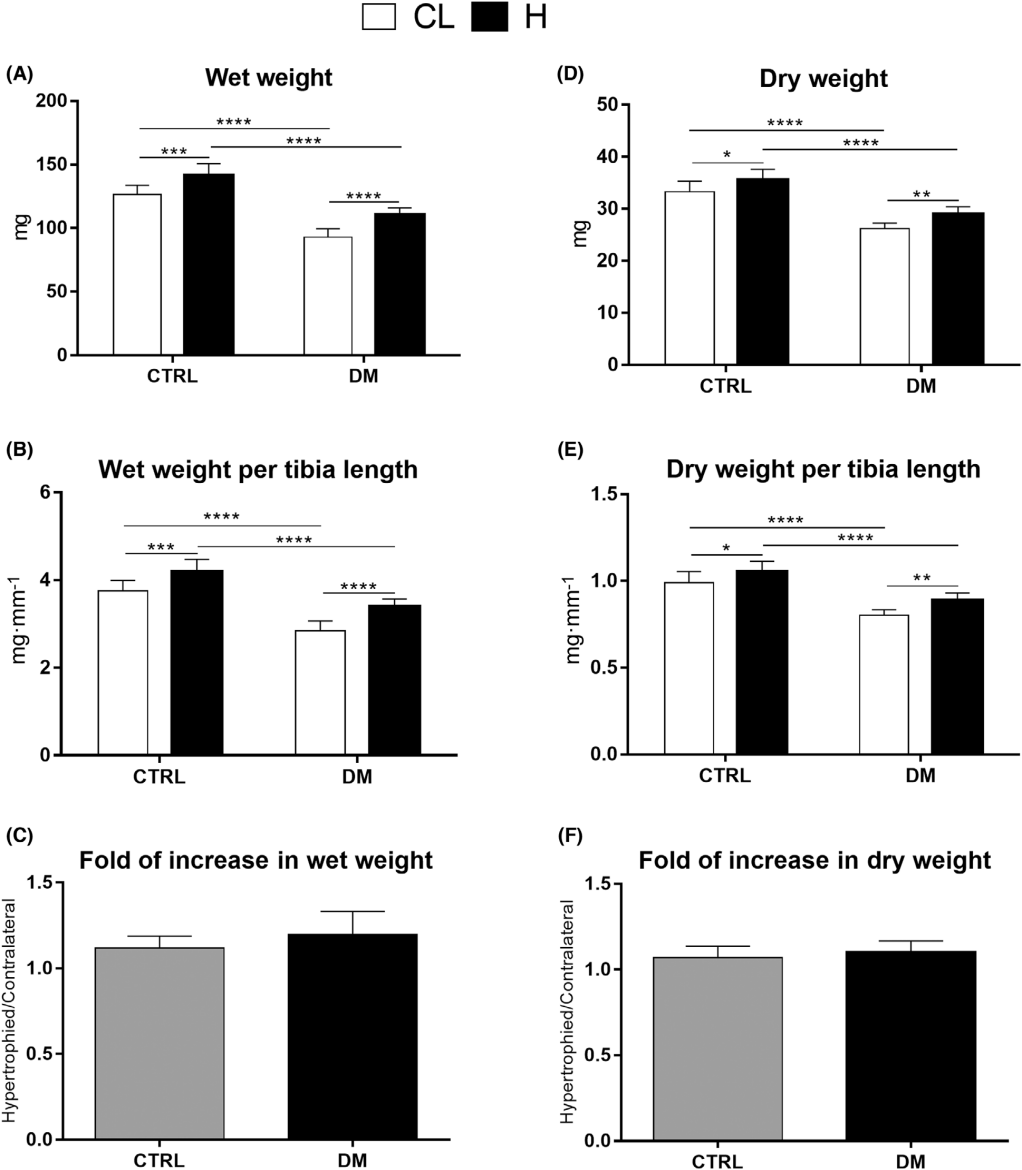
Mass of extensor digitorum longus (EDL) muscles from control (CTRL) and diabetic (DM) rats after 7 days of overload. EDL muscle wet mass (A). EDL muscle wet mass normalized to tibial length (B). Ratio of wet mass of hypertrophied (H) and contralateral (CL) EDL muscles (C). Dry mass of the EDL muscle (D). Dry mass of EDL muscle normalized to tibial length (E). Ratio of the dry mass of hypertrophied (H) and contralateral (CL) EDL muscles (F). *P < 0.05; **P < 0.01; ***P < 0.001; ****P < 0.0001 as indicated by two‐way ANOVA followed by Bonferroni post‐test. Results of panels C and F were analyzed using Student's t‐test. Values are expressed as the mean ± SD. The number of animals used in each group was: CTRL = 10 and DM = 8.

**Fig. 4:**
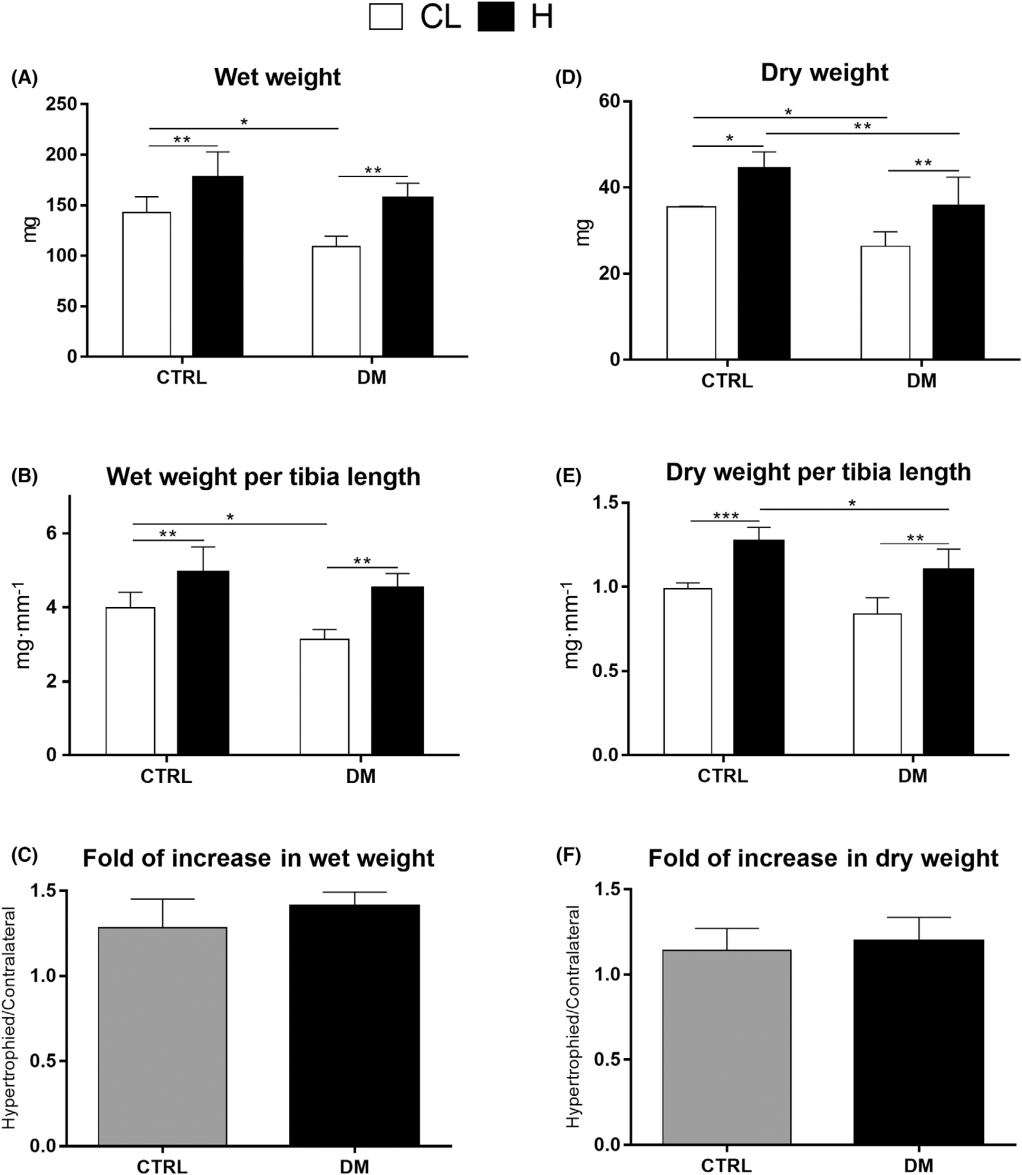
Mass of soleus muscles from control (CTRL) and diabetic (DM) rats after 7 days of overload. Wet mass of soleus muscle (A). Wet mass of soleus muscle normalized to tibial length (B). Ratio of wet mass of hypertrophied (H) and contralateral (CL) soleus muscles (C). Dry mass of the soleus muscle (D). Dry mass of soleus muscle normalized to tibial length (E). Ratio of the dry mass of hypertrophied (H) and contralateral (CL) soleus muscles (F). *P < 0.05; **P < 0.01; ***P < 0.001 as indicated by two‐way ANOVA followed by Bonferroni post‐test. Results of panels C and F were analyzed by Student's *t*‐test. Values are expressed as the mean ± SD. The number of animals used in each group was: CTRL = 4 and DM = 4.

The wet and dry weights of EDL and soleus muscles of diabetic rats were significantly lower than those of control animals (Figs 3 and 4, panels A and D), even after normalization to tibial length (Figs 3 and 4, panels B and E). Diabetes decreased the dry weight of contralateral EDL muscles by 22%. After 7 days of overload‐induced hypertrophy, EDL muscle dry weight increased by 7% in control and 10% in diabetic rats (Fig. 3, panel D). Under the same conditions, the dry weight of the soleus muscle increased by 28% and 31% in control and diabetic rats (Fig. 4, panel D). The increase in hypertrophied muscle wet and dry weights, normalized to tibial length, compared with the contralateral muscle, was similar in both groups (Figs 3 and 4, panels C and F). Thus, both groups' EDL and soleus muscles displayed similar hypertrophic responses.

**Fig. 5:**
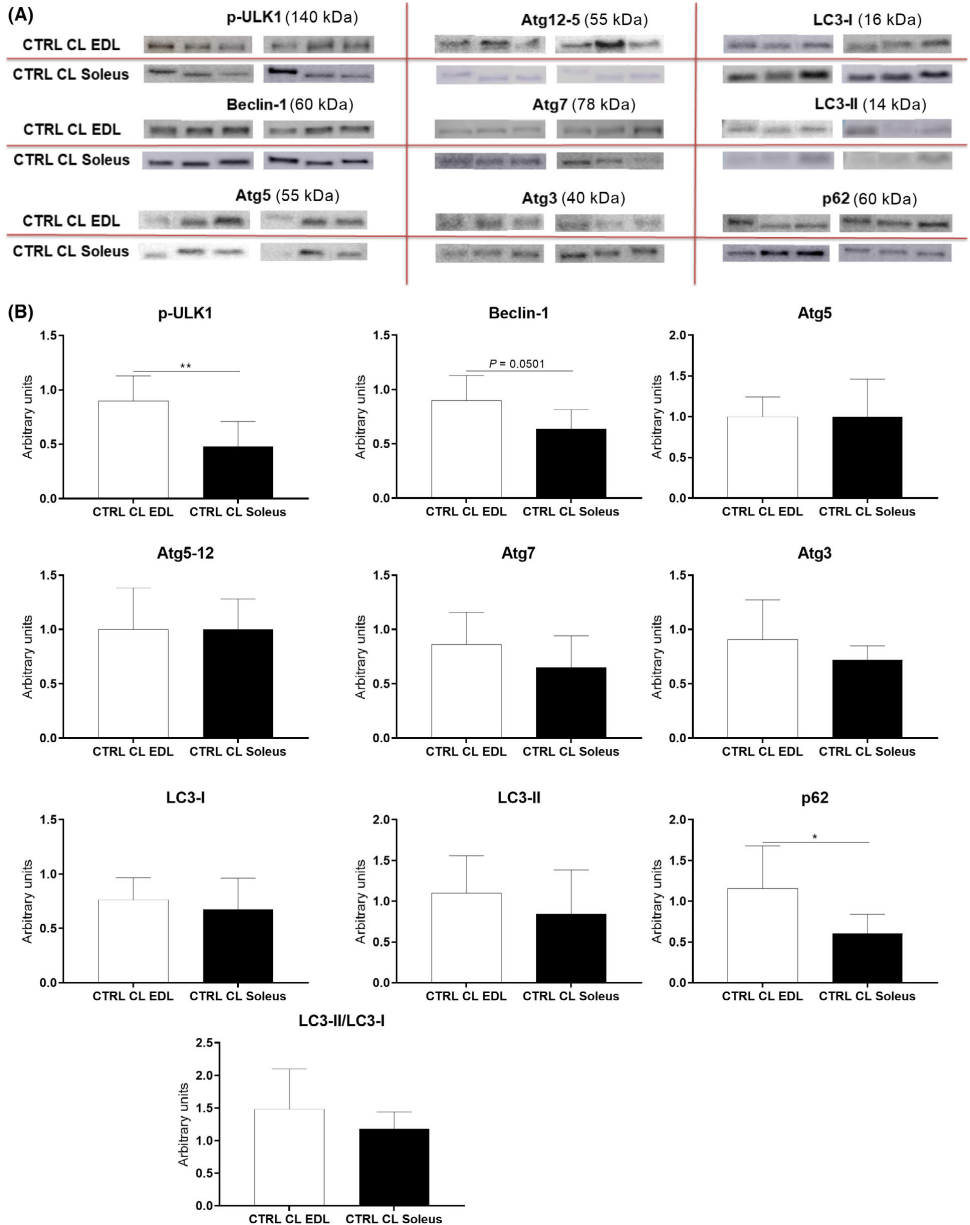
Representative protein blots (A) and levels of autophagy signaling proteins (B) in extensor digitorum longus (EDL) and Soleus muscles of control rats (CTRL) before overload (CL), measured by western blotting. The following proteins were measured: p‐ULK1, Beclin‐1, Atg5, Atg12‐5, Atg7, Atg3 LC3‐I, LC3‐II, p62/SQSTM1, and LC3‐II /LC3‐I ratio. Each band presented in panel A was extracted from the original gel, and all their intensities were normalized by the respective Ponceau S. All raw data are exhibited in Appendix S1 (Attachment 1 for EDL and 2 for Soleus). Statistical analysis was performed using the Student's *t*‐test; *P < 0.05; **P < 0.01. Values are expressed as the mean ± SD. Six animals were used per group. CTRL CL EDL = Contralateral EDL muscle of control animal; CTRL CL Soleus = Contralateral Soleus muscle of control animal. The number of animals used in each group was 6.

Next, we assessed autophagic signaling protein levels (p‐ULK1, Beclin‐1, Atg5, 12–5, 7 and 3, p62/SQSTM1, LC3‐I and LC3‐II, and the LC3‐II/LC3I ratio) in the EDL and soleus muscles of control and diabetic rats before and after overload‐induced hypertrophy. As shown in Fig. 5, the EDL muscle had augmented levels of p‐ULK1 (89%), Beclin‐1 (40%), and p62/SQSTM1 (91%) compared with the soleus muscle.

**Fig. 6:**
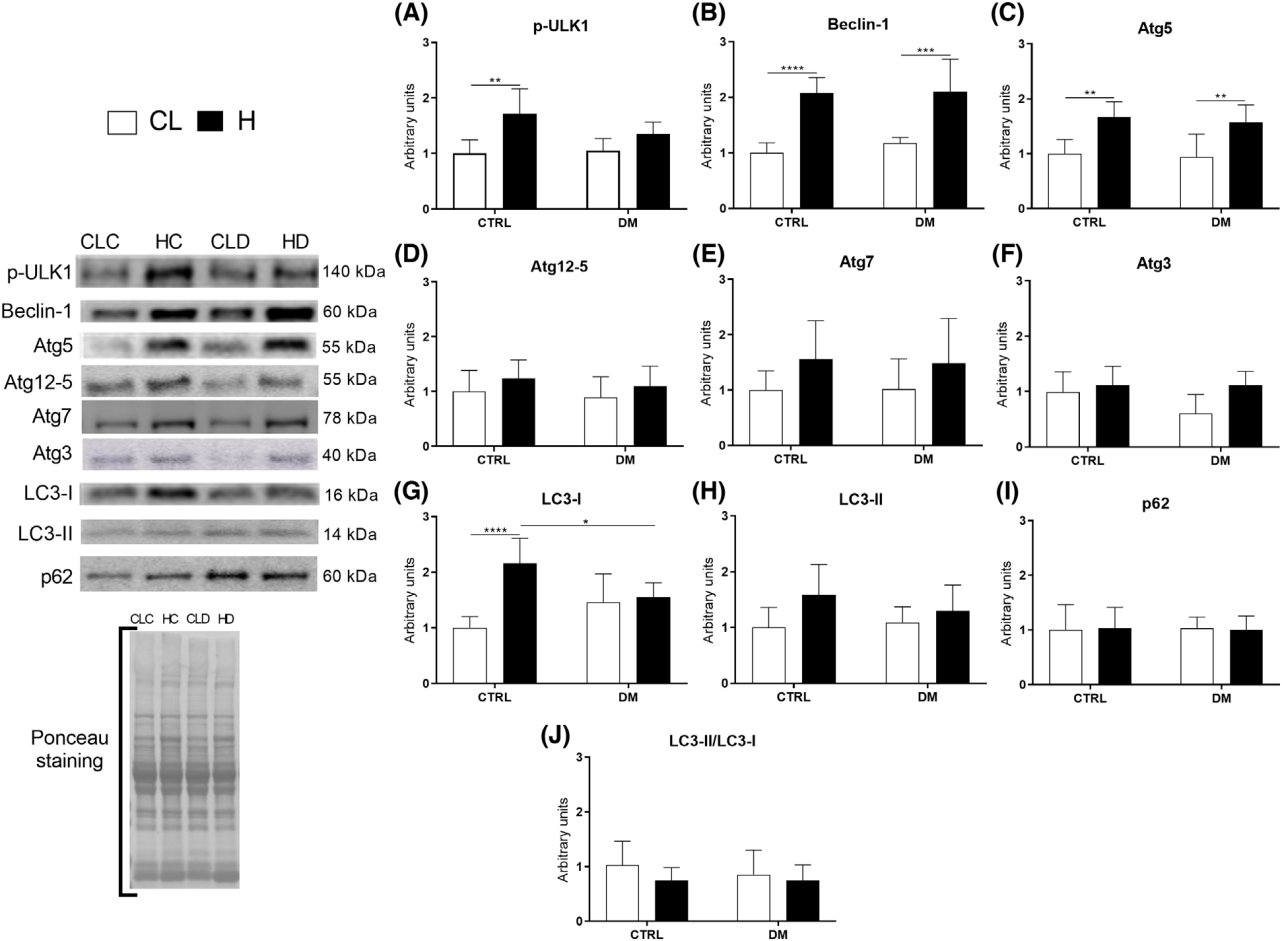
Autophagy signaling protein levels in extensor digitorum longus (EDL) muscles of control (CTRL) or diabetic (DM) rats after 7 days of overload‐induced hypertrophy, measured by western blotting. Representative protein blots stained with Ponceau S are inserted. The following proteins were measured: p‐ULK1 (A), Beclin‐1 (B), Atg5 (C), Atg12‐5 (D), Atg7 (E), Atg3 (F) LC3‐I (G), LC3‐II (H), p62/SQSTM1 (I), and LC3‐II /LC3‐I ratio using the results in panels G and H (J). Statistical analysis was performed using two‐way ANOVA followed by Bonferroni post‐test; *P < 0.05; **P < 0.01; ***P < 0.001; ****P < 0.0001. Values are expressed as the mean ± SD. Each group was composed of six animals. CL, contralateral muscle; CLC, control group contralateral muscle; CLD, diabetic group contralateral muscle; H, hypertrophied muscle; HC, control group hypertrophied muscle; HD, diabetic group hypertrophied muscle.

**Fig. 7:**
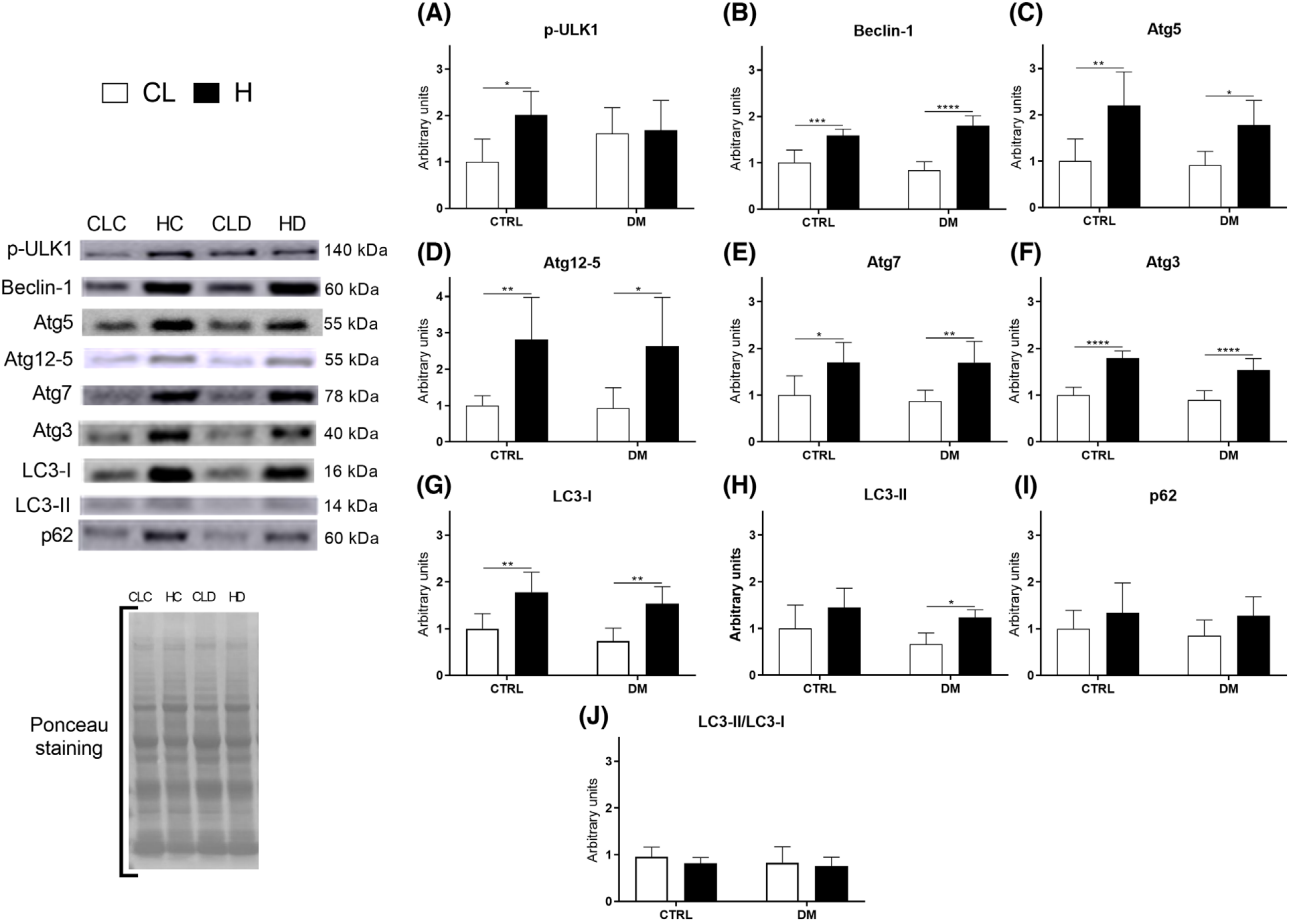
Autophagy signaling protein levels in soleus muscles of control (CTRL) or diabetic (DM) rats after 7 days of overload‐induced hypertrophy, measured by western blotting. Representative protein blots stained with Ponceau S are inserted. The following proteins were measured: p‐ULK1 (A), Beclin‐1 (B), Atg5 (C), Atg12‐5 (D), Atg7 (E), Atg3 (F) LC3‐I (G), LC3‐II (H), p62/SQSTM1 (I), and LC3‐II /LC3‐I ratio using the results in panels G and H (J). Statistical analysis was performed using two‐way ANOVA followed by Bonferroni post‐test; *P < 0.05; **P < 0.01; ***P < 0.001; ****P < 0.0001. Values are expressed as the mean ± SD. Each group was composed of 6 animals. CL, contralateral muscle; CLC, control group contralateral muscle; CLD, diabetic group contralateral muscle; H, hypertrophied muscle; HC, control group hypertrophied muscle; HD, diabetic group hypertrophied muscle.

The autophagy signaling protein levels were also measured after 7 days of overload in the EDL (Fig. 6) and soleus (Fig. 7) muscles of control and diabetic rats.

Overload had marked effects on autophagy signaling protein levels of EDL muscle. For example, p‐ULK1 content increased by 71% in the control group compared with the contralateral muscle, but it did not change in diabetic rats (Fig. 6, panel A). Beclin‐1 content increased twofold in the control group and 79% in the diabetic group (Fig. 6, panel B). Additionally, Atg5 content was increased by 67% and 68% in control and diabetic rats, respectively (Fig. 6, panel C). By contrast, Atg12‐5 (Fig. 6, panel D), Atg7 (Fig. 6, panel E), Atg3 (Fig. 6, panel F), and p62 (Fig. 6, panel I) were not significantly changed in the EDL muscle of either group. LC3‐I content increased twofold in control but remained unchanged in diabetic rats (Fig. 6, panel G). Moreover, LC3‐II content in the hypertrophied muscle of control rats was 39% higher than in diabetic ones (Fig. 6, panel H). However, none of the LC3‐I and LC3‐II alterations significantly altered the ratios of the two proteins (Fig. 6, panel J).

**Fig. 8:**
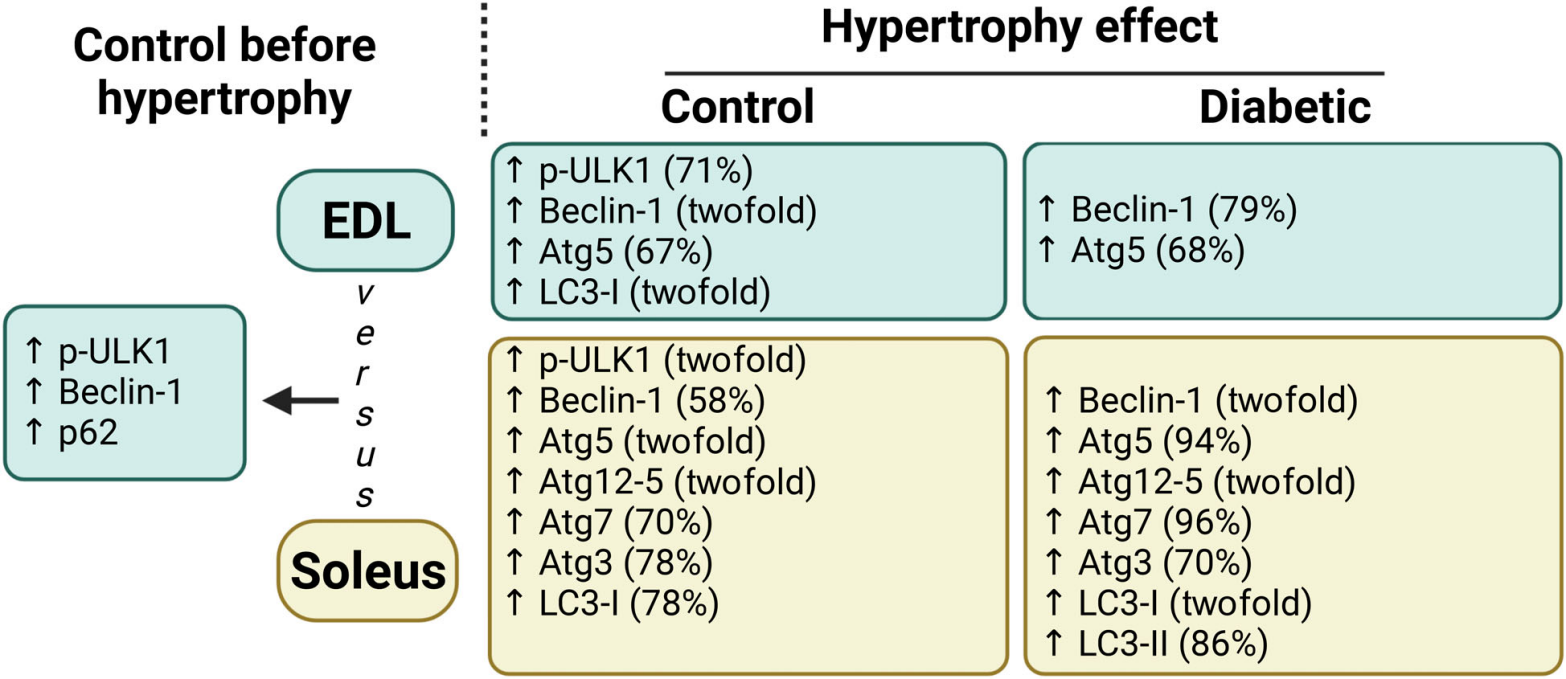
Summary of extensor digitorum longus (EDL) and soleus muscle results after 7 days of functional overload (only statistically significant results are reported). Neither diabetes nor overload‐induced muscle hypertrophy markedly changed the expression of Atg12‐5, Atg3, Atg7, LC3‐II, and p62/SQSTM1 or the LC3‐II/LC3‐I ratio in EDL muscle. Neither diabetes nor overload‐induced muscle hypertrophy changed the p62/SQSTM1 expression or the LC3‐II/LC3‐I ratio in the soleus muscle.

Overload induced more pronounced changes in autophagy signaling protein levels in the soleus than detected in the EDL. As shown in Fig. 7, the p‐ULK1 content was increased by twofold in control rats (Fig. 7, panel A), Beclin‐1 increased by 58% in the control group and twofold in the diabetic one (Fig. 7, panel B), Atg5 increased by twofold in the control and 94% in the diabetic group (Fig. 7, panel C), Atg12‐5 increased by twofold in control and diabetic rats (Fig. 7, panel D), Atg7 increased by 70% in control and 96% in diabetic rats (Fig. 7, panel E), Atg3 increased by 78% in the control and 70% in the diabetic groups (Fig. 7, panel F), LC3‐I increased by 78% in the control and twofold in the diabetic groups (Fig. 7, panel G), and LC3‐II increased by 86% in the diabetic group but did not change in control rats (Fig. 7, panel H). Like the EDL muscle results, p62 (Fig. 7, panel I) and the LC3‐II/LC3‐I ratio (Fig. 7, panel J) were not altered. A summary of the results from Figs 6 and 7 is presented in Fig. 8.

## Discussion

Our study is the first to evaluate autophagy signaling protein expression 7 days post‐overload‐induced hypertrophy in the EDL and soleus muscles of control and T1DM rats. It was previously reported that 7 days of the diabetic state attenuates the EDL skeletal muscle mass [18, 36, 37], an observation consistent with the present study. On the contrary, the short diabetic state duration did not affect the soleus muscle mass. It has been proposed that this differential response is due to oxidative muscle fibers (soleus) being more resistant to functional impairment and muscle mass loss in the diabetic state than glycolytic ones (EDL) [18, 38, 39]. Despite the impairment of muscle mass by T1DM, streptozotocin‐induced diabetes concomitant with EDL or soleus muscle overload did not alter hypertrophy after 7 days. In our previous study, the diabetic rats showed a similar hypertrophic response in the same muscles as the control group [17, 18].

It has been proposed that autophagy plays a role in maintaining skeletal muscle homeostasis [11, 40], and the EDL muscle had higher basal levels of p‐ULK1, Beclin‐1, and p62/SQSTM1 compared with the soleus before hypertrophy induction. Interestingly, Paré et al. [41] reported that autophagic signaling protein levels are lower in a glycolytic muscle than in an oxidative one; however, the glycolytic muscle's basal autophagic flux was augmented.

Several studies demonstrated that T1DM exposes skeletal muscle to signals that could alter autophagy activity [42, 43, 44, 45]. However, in the present study, the diabetic state *per se* did not affect autophagy signaling protein levels in either muscle compared with control animals. In contrast, others reported a diabetes‐induced increase in autophagy signaling proteins in the gastrocnemius [46, 47], soleus, and EDL muscles of mice after 9–10 weeks of diabetes [48, 49]. In this sense, the duration of the diabetic state may influence autophagy‐related protein expression.

Under basal conditions, specific autophagy signaling protein levels were higher in the EDL muscle. However, the soleus muscle had a more substantial increase in autophagic signaling protein levels after overload‐induced hypertrophy, which were also elevated in diabetic animals (Figs 6 and 7). Previous studies showed that overload‐induced skeletal muscle hypertrophy stimulates autophagy [50, 51, 52]. Indeed, muscle contraction increases protein synthesis and generates reactive oxygen species, enhancing the need for autophagic clearance of damaged cellular components to maintain the working muscle mass and optimal muscle protein content [53]. Moreover, autophagy provides energy for the cells to sustain muscle cell integrity and function [4, 54].

In the baseline condition (without diabetes and hypertrophy), our results showed that EDL muscle exhibited greater autophagy, as indicated by the upregulation of phospho‐ULK1, Beclin‐1, and p62 proteins. After the compensatory hypertrophy protocol, there was an increase in autophagic markers associated with increased protein turnover, ultimately leading to a net positive effect. The mentioned increase was observed in the EDL muscle of control animals (increases in p‐ULK1, Beclin‐1, Atg5, and LC3‐I) and diabetic animals (increases in Beclin‐1 and Atg5), and in the soleus muscle of the control group (increase in p‐ULK1, Beclin‐1, Atg5, Atg12‐5, Atg7, Atg3, and LC3‐I) and diabetic animals (increase in Beclin‐1, Atg5, Atg12‐5, Atg7, Atg3, and LC3‐I and LC3‐II).

The protein ULK1 is activated by AMPK (protein kinase activated by AMP) due to a negative ATP balance in the absence of nutrients and is inhibited by mTOR activation [55]. After 7 days of overload‐induced hypertrophy, p‐ULK1 content increased in the control rats and remained unchanged in the diabetic ones. Nevertheless, the p‐ULK1 discrepancy had a negligible effect on the upregulation of other downstream autophagy signaling protein levels caused by overload‐induced hypertrophy in both groups.

Beclin‐1, which increased after hypertrophy in control and diabetic animals, acts in autophagosome formation at the beginning of the process and can be activated via the ULK1‐dependent pathway [56] or through phosphorylation and subsequent inhibition of Bcl‐2 (B‐cell lymphoma 2), which uncouples from Beclin‐1 and promotes its activation under conditions of muscle activity [4, 57].

The Atg12‐5‐16L complex is required for membrane transport to its target and the lipidation of LC3‐I to form LC3‐II [58]. Raben et al. [15] reported that silencing the Atg5 gene results in skeletal muscle mass loss, protein aggregation, abnormal membrane structure accumulation, and impaired muscle strength. Additionally, Atg5 transgenic mice moderately overexpressing this protein show enhanced autophagy and prolonged mean lifespan [59]. We found that Atg5 alone significantly increased after hypertrophic stimulation in both groups. On the contrary, only Atg12‐5 increased in the soleus muscle following overload.

It is known that Atg7 plays a role in holding the Atg12‐5‐16L complex together [60]. Knockdown of Atg7 leads to myopathy, misalignment of the A and Z bands, and increased numbers of mitochondria with membranous structures and protein aggregates. The absence of Atg7 also leads to a 20–40% reduction in skeletal muscle cross‐sectional area [14]. At the same time, overexpression of Atg7 in aged mice restores the loss of neuromuscular function and autophagy activity due to aging. Herein, Atg7 levels increased after soleus muscle hypertrophy in both groups.

In the intermediate phase of the autophagic process, Atg7, together with Atg3, conjugates phosphatidylethanolamine in the LC3‐I molecule [60], producing LC3‐II, which is the actual effector molecule of the autophagic process. Atg3 knockout mice exhibit disrupted autophagosome formation due to a defect in this conjugation step, which prevents the closure of the isolation membrane [61]. We observed an increased Atg3 response in hypertrophied soleus muscle of diabetic and control rats.

Furthermore, we observed upregulation of LC3‐I due to hypertrophy in the EDL and soleus muscles of the control group. The soleus muscle of diabetic rats exhibited increased LC3‐I and LC3‐II content only after hypertrophy. It is important to point out that the LC3‐II/LC3‐I ratio is used to represent autophagic flux [62], and no changes in this parameter were detected in the present study.

In the final steps of autophagy, LC3‐II is coupled to the autophagosome membrane, and the p62/SQSTM1 acts as a bridge between ubiquitinated substrates bound to the cargo destined to be degraded and LC3‐II, finally promoting the closure of the isolation membrane and degradation. We did not observe changes in p62/SQSTM1 levels in either muscle or experimental group after the overload. A previous study demonstrated that once the autophagosome encloses and fuses with the lysosome, p62/SQSTM1 levels are attenuated due to autolysosomal degradation, thus making it difficult to detect this protein [63].

A limitation of this study is the lack of a paired feeding DM group. However, the aim of this study was achieved since we compared a well‐established diabetic state with a control condition, and novel discoveries were reported.

## Conclusions

The EDL muscle had higher autophagy signaling protein levels at the baseline. By contrast, the soleus muscle exhibited more elevated autophagy signaling protein levels in control and diabetic rats after hypertrophy induction. The magnitude of a 7‐day compensatory overload‐induced hypertrophy of EDL and soleus muscles of control and three‐day diabetic rats was not different. Diabetes did not alter autophagy signaling protein levels of EDL and soleus muscles before or after functional load hypertrophy.

## Conflict of interest

The authors declare no conflict of interest.

### Peer review

The peer review history for this article is available at https://www.webofscience.com/api/gateway/wos/peer‐review/10.1002/2211‐5463.13677.

## Author contributions

MVMS conceived the study, conducted molecular biology experiments, and wrote the manuscript. MASF and EBS assisted in the molecular biology experiments, statistical analysis, animal surgeries, and interpretation of the results. KFV and ACL‐P revised the manuscript for important intellectual content. GMM assisted in the molecular biology experiments. DRS, GOS, ACL‐P, WMTK, and TCAL interpreted and discussed the results. RC conceived the study, revised the manuscript for important intellectual content, assisted in the statistical analysis, and drafted the manuscript. All authors read and approved the final manuscript version.

## Supporting information


**Appendix S1.** Supplemental material from western blots of the EDL and soleus muscles.

## Data Availability

The authors declare the availability of supporting data contained within the manuscript (such as Appendix S1).
