# Supplementary material for: Autophagy signaling in hypertrophied muscles of diabetic and control rats
Source: FEBS Open Bio. 2023 Jul 26;13(9):1709–22. doi: 10.1002/2211-5463.13677 (PMC10476571; doi:10.1002/2211-5463.13677)
Supplement: Supplementary file 1 — Appendix S1. Supplemental material from western blots of the EDL and soleus muscles. [file FEB4-13-1709-s001.pdf]

Supplementary Information

Attachment 1: Blots used to measure protein changes in expression of p-ULK1 (A), Beclin-1 (B), Atg5 (C), Atg12-5 (D), Atg7 (E), Atg3 (F), LC3-I and II (G), and p62 (H) in contralateral and hypertrophied extensor digitalis longus (EDL) muscles of control and diabetic rats (presented in Figure 6). Gels obtained by western blotting with their respective total protein content (evaluated by Ponceau S) in EDL muscles after seven days of functional overload. Six animals were used per group. CLC - contralateral muscle of the control group; HC - Hypertrophied muscle of the control group; CLD - Muscle contralateral of the diabetic group; HD - Hypertrophied muscle of the diabetic group.

A

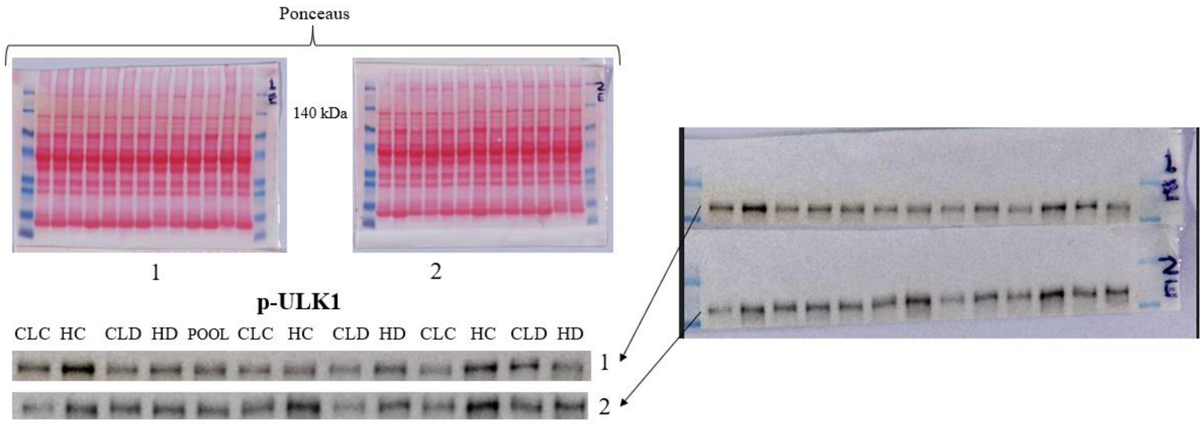

B

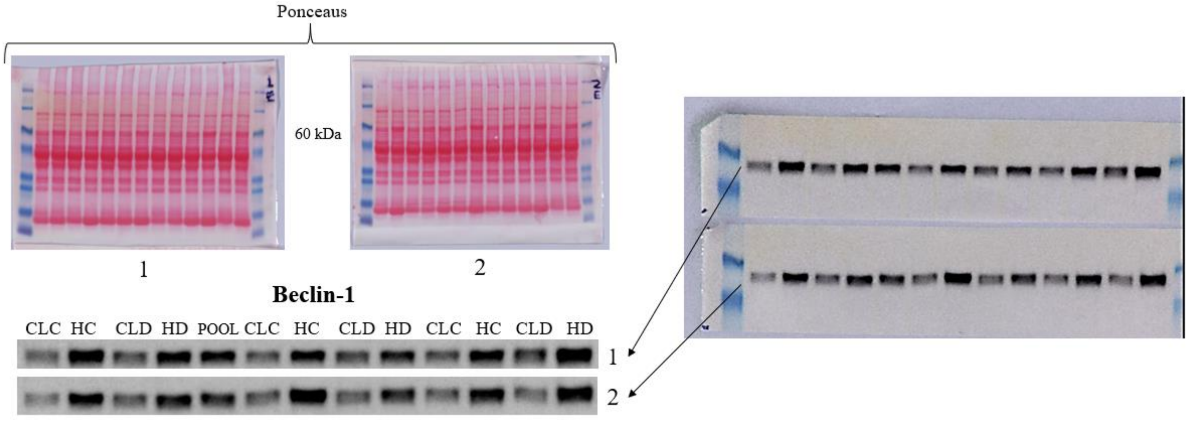

C

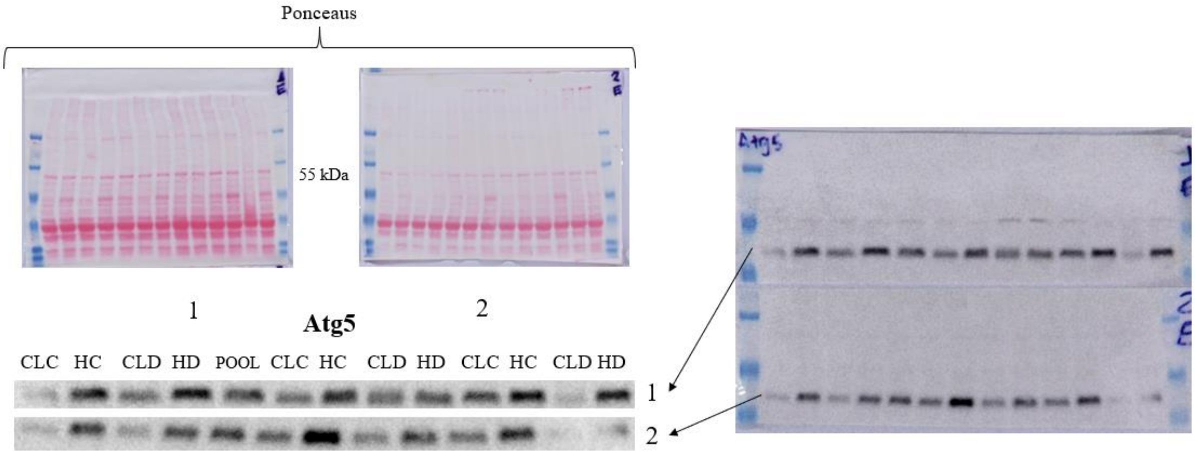
Ponceaus
55 kDa
1
2
Atg5
CLC HC CLD HD POOL CLC HC CLD HD CLC HC CLD HD
1
2

D

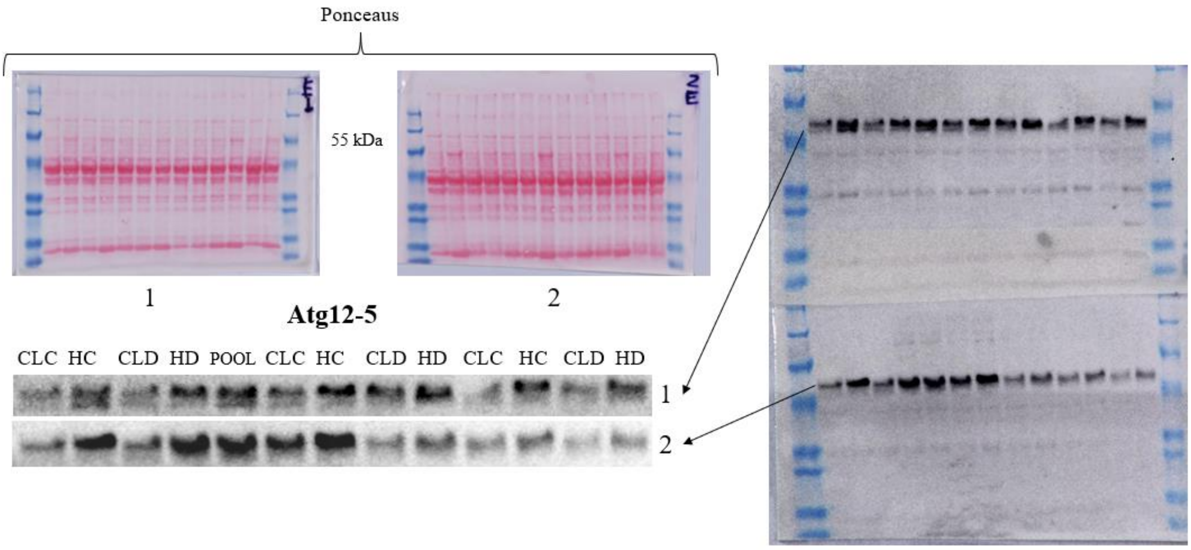
Ponceaus
55 kDa
1
2
Atg12-5
CLC HC CLD HD POOL CLC HC CLD HD CLC HC CLD HD
1
2

E

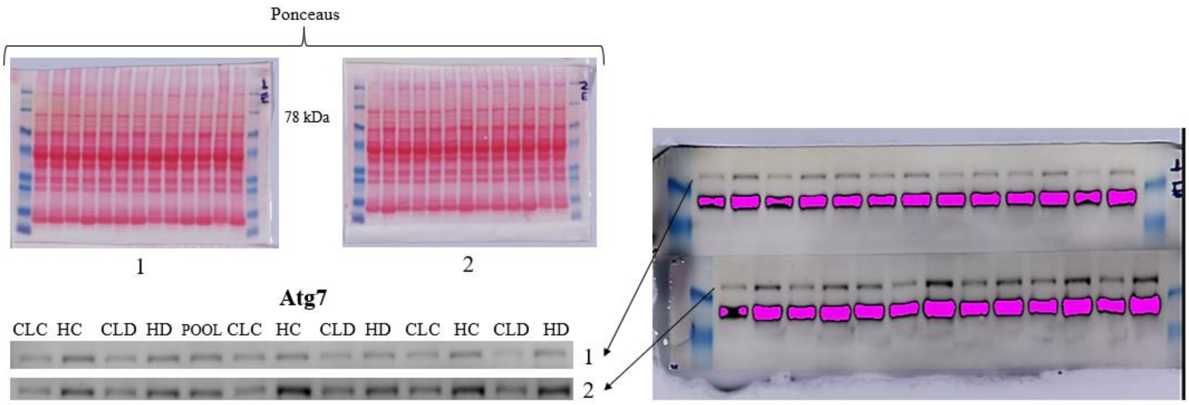
Ponceaus
78 kDa
1
2
Atg7
CLC HC CLD HD POOL CLC HC CLD HD CLC HC CLD HD
1
2

F

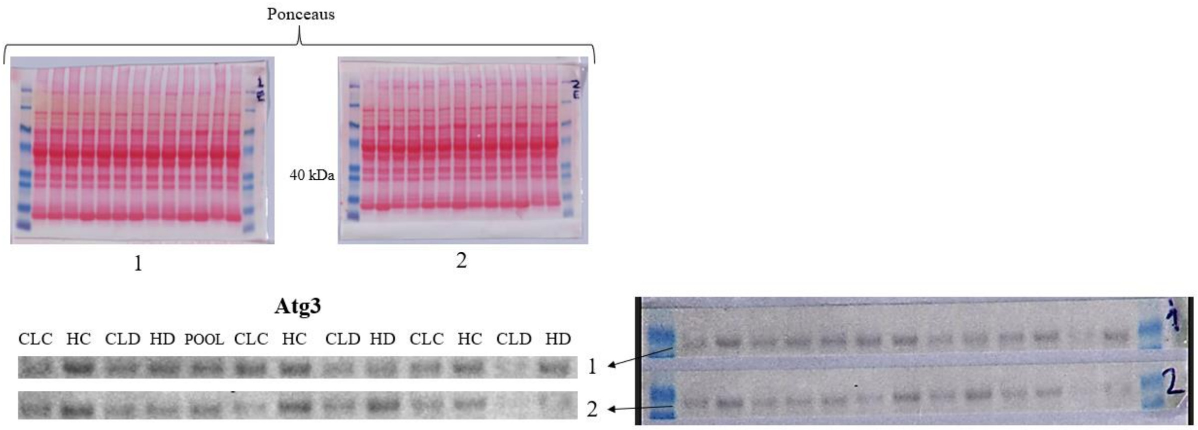
Ponceaus
40 kDa
1
2
Atg3
CLC HC CLD HD POOL CLC HC CLD HD CLC HC CLD HD
1
2

G

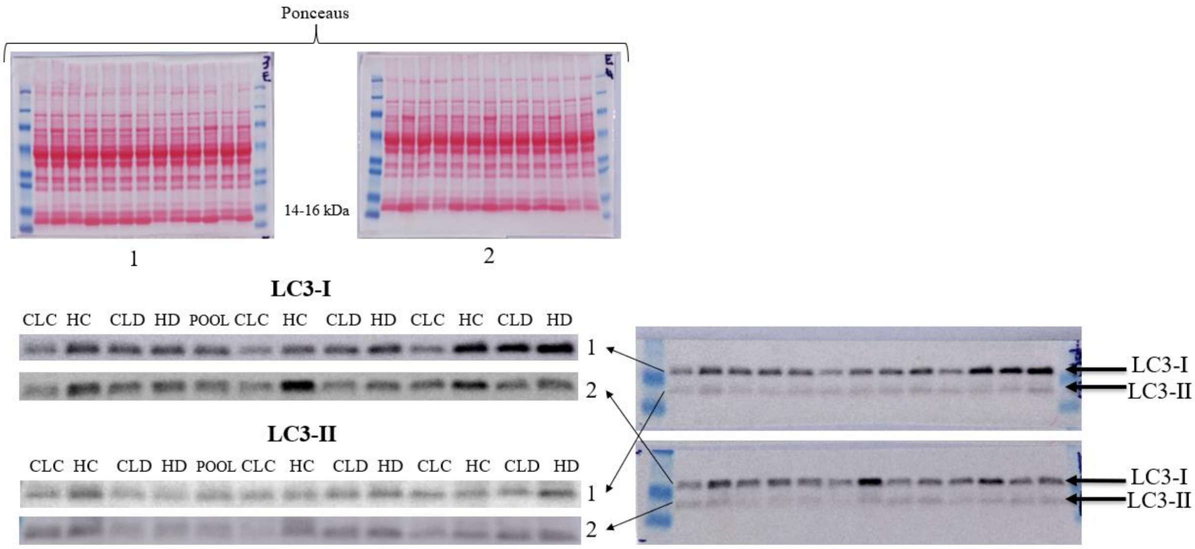
Ponceaus
14-16 kDa
1
2
LC3-I
CLC HC CLD HD POOL CLC HC CLD HD CLC HC CLD HD
1
2
LC3-II
CLC HC CLD HD POOL CLC HC CLD HD CLC HC CLD HD
1
2
LC3-I
LC3-II
LC3-I
LC3-II

H

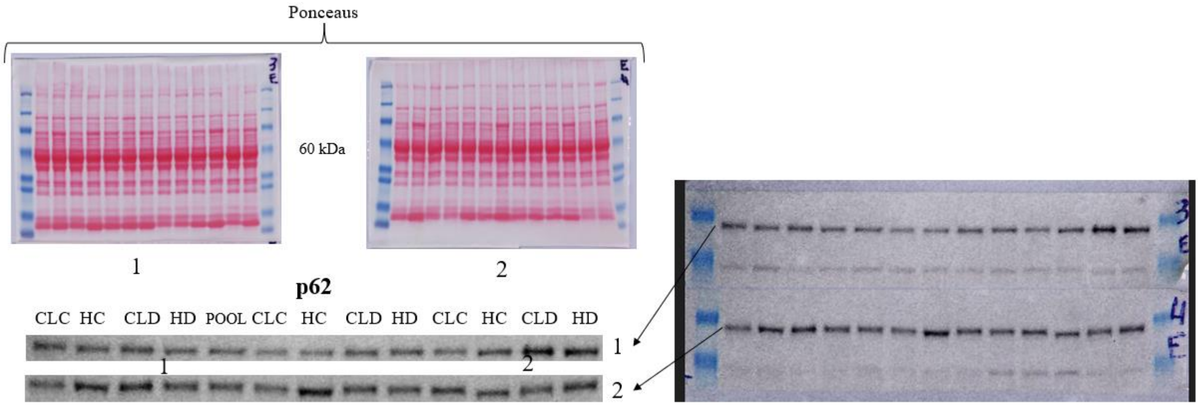
Ponceaus
60 kDa
1
2
p62
CLC HC CLD HD POOL CLC HC CLD HD CLC HC CLD HD
1
2

Attachment 2: Blots used to measure protein changes in expression of p-ULK1 (A), Beclin-1 (B), Atg5 (C), Atg12-5 (D), Atg7 (E), Atg3 (F), LC3-I and II (G), and p62 (H) in contralateral and hypertrophied soleus muscles of control and diabetic rats (presented in Figure 7). Gels obtained by western blotting with their respective total protein content (evaluated by Ponceau S) in EDL muscles after seven days of functional overload. Six animals were used per group. CLC - contralateral muscle of the control group; HC - Hypertrophied muscle of the control group; CLD - Muscle contralateral of the diabetic group; HD - Hypertrophied muscle of the diabetic group.

A

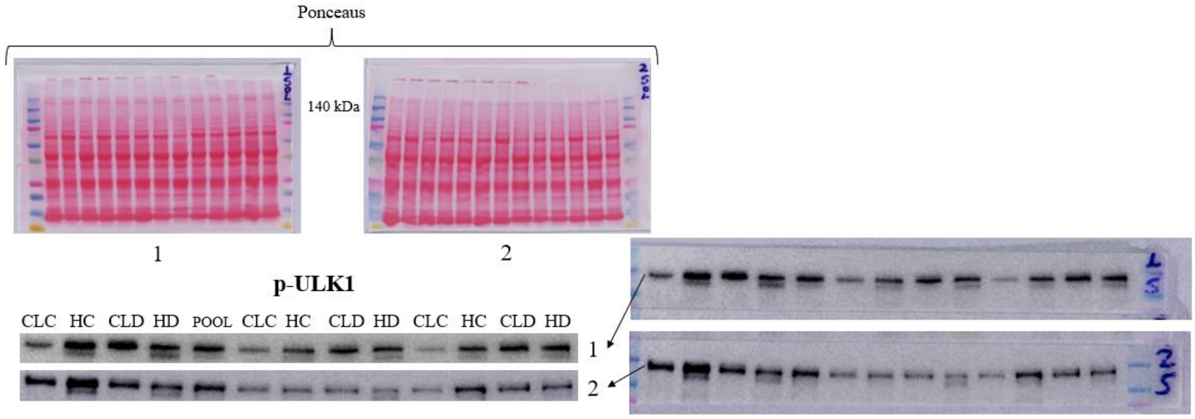

B

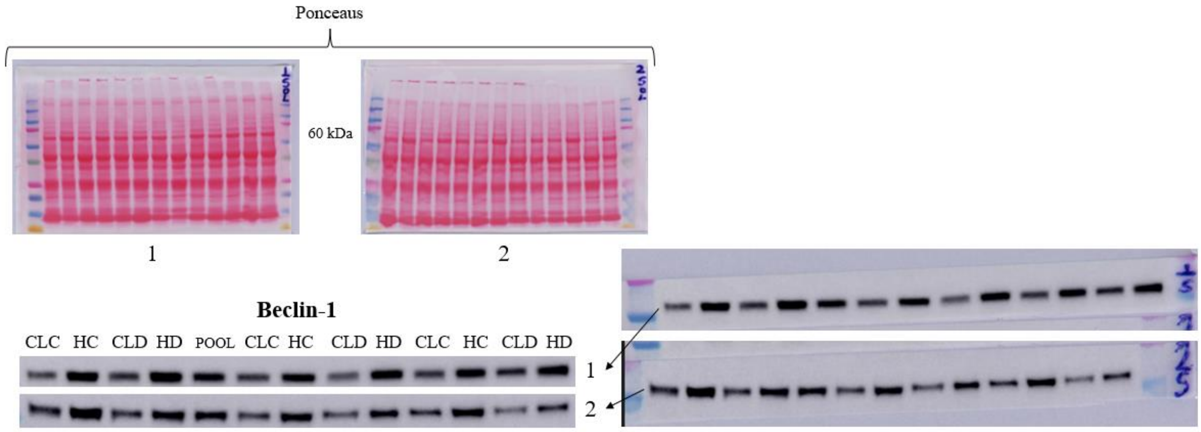

C

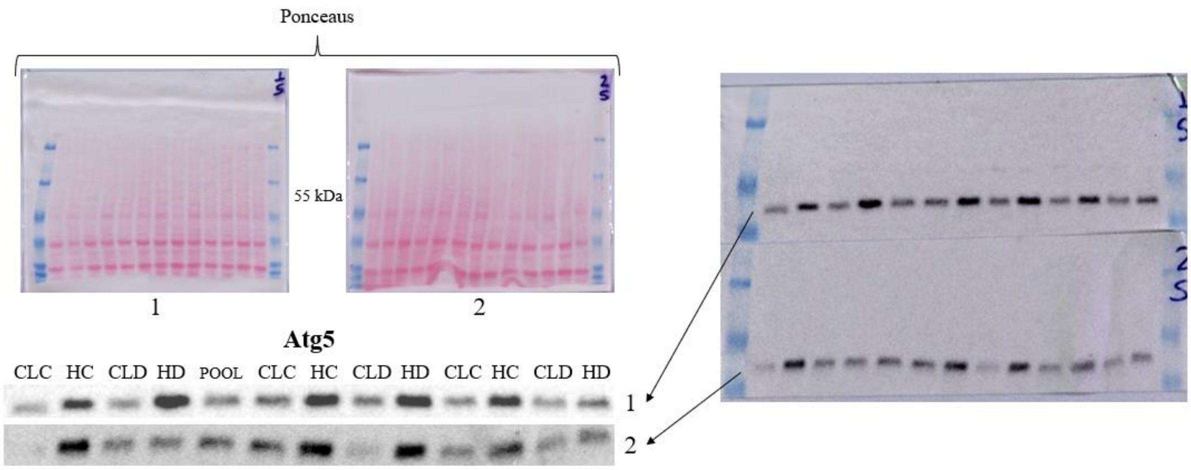
Ponceaus
55 kDa
1
2
Atg5
CLC HC CLD HD POOL CLC HC CLD HD CLC HC CLD HD
1
2

D

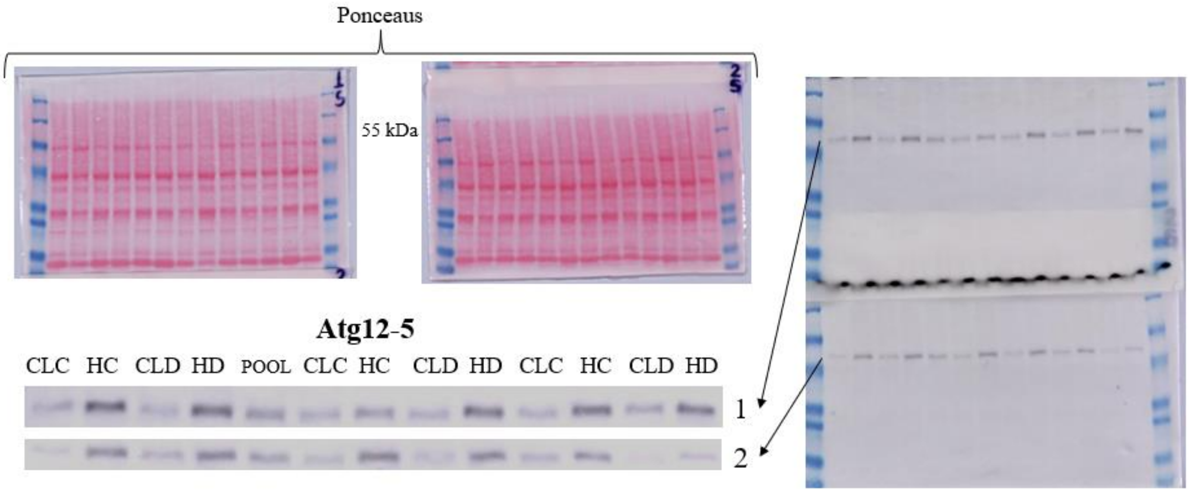
Ponceaus
55 kDa
Atg12-5
CLC HC CLD HD POOL CLC HC CLD HD CLC HC CLD HD
1
2

E

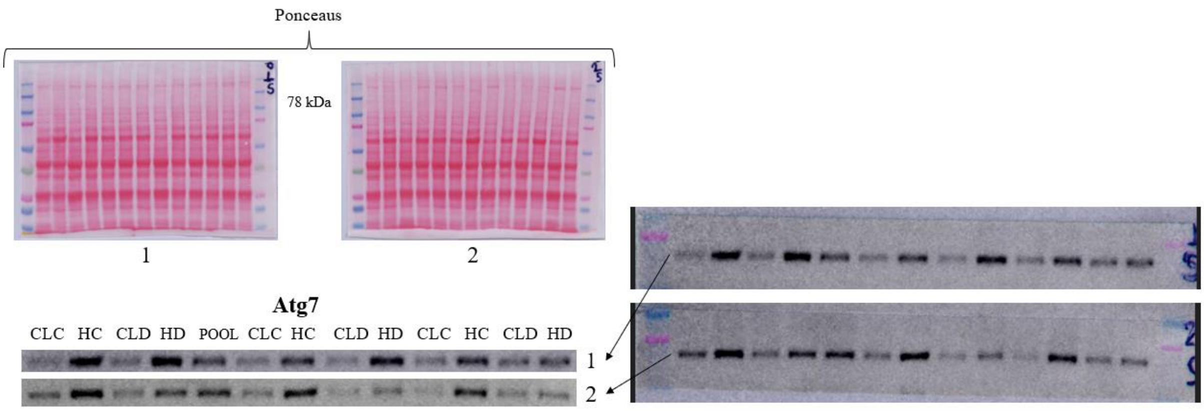
Ponceaus
78 kDa
1
2
Atg7
CLC HC CLD HD POOL CLC HC CLD HD CLC HC CLD HD
1
2

F

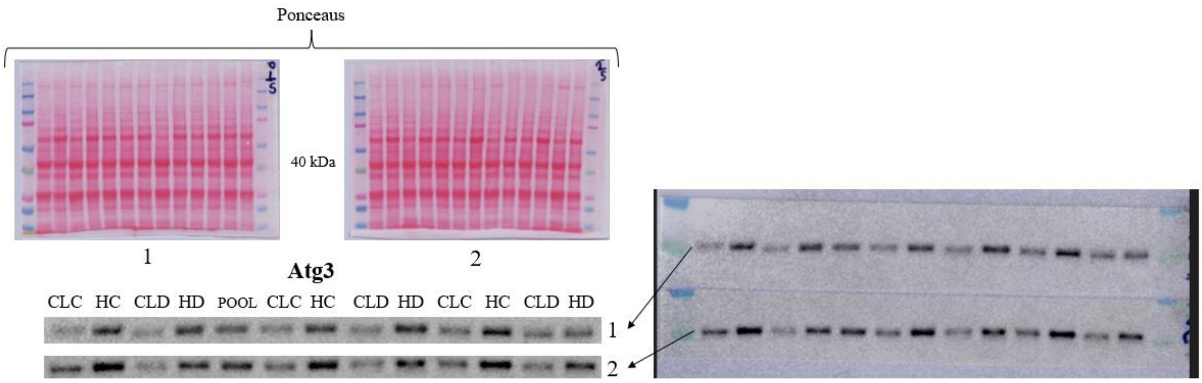
Ponceaus
40 kDa
1
2
Atg3
CLC HC CLD HD POOL CLC HC CLD HD CLC HC CLD HD
1
2

G

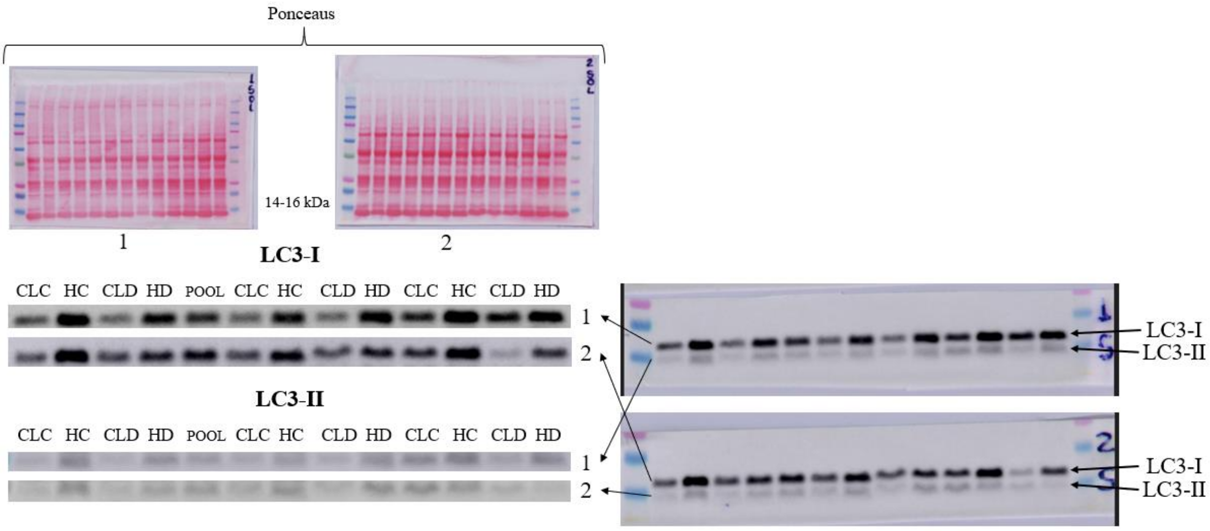
Ponceaus
14-16 kDa
1
2
LC3-I
CLC HC CLD HD POOL CLC HC CLD HD CLC HC CLD HD
1
2
LC3-II
CLC HC CLD HD POOL CLC HC CLD HD CLC HC CLD HD
1
2
LC3-I
LC3-II
LC3-I
LC3-II

H

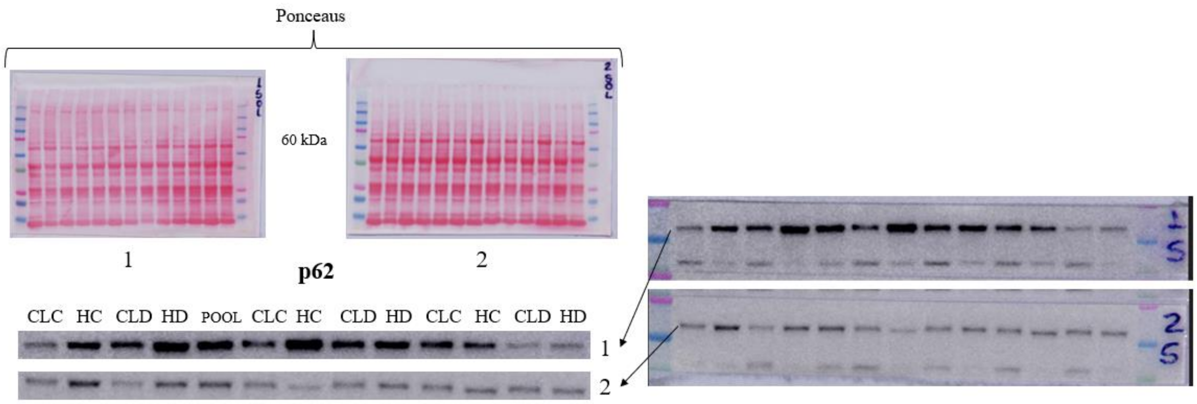
Ponceaus
60 kDa
1
2
p62
CLC HC CLD HD POOL CLC HC CLD HD CLC HC CLD HD
1
2
